# Microscopy of the Dental Tissues. New Series

**Published:** 1869-11

**Authors:** S. P. Cutler


AMERICAN JOURNAL

OF

DENTAL SCIENCE.

Vol. III. THIRD SERIES--NOVEMBER 1869. No. 7.

### ARTICLE I.

### *Microscopy of the Dental Tissues. New Series.*

By S. P. CUTLER, M.D., D.D.S.

*Continued.*

*Physiology, Histology, and Pathology.*—In my last article I spoke of the physiology of muscularity, I stated that by that mysterious power or force called volition, muscles of volition responded by contraction when the volite charge was turned loose into such muscles by letting loose the trigger that held such force in static abeyance.

When this force is let loose and muscle contracts, antagonizing muscle relaxes from its state of pressive tension in consonance with muscle contracted, and continues so long as contraction continues, and when contracted muscle relaxes the former balancing equipoise is resumed, the relaxed muscle now contracts slightly, and formerly contracted muscle now, in turn, relaxes, and both fall into their normal state of rest, tension now being equal. In the function of contraction and relaxation there was formed or existed a cycle and afterwards another cycle corresponding to the pre-existing cycle. In this muscular contraction there must have been a double current, one, the positive, causing contraction, and the most energetic, the other, causing relaxation, negative or weaker. One afferent and the other efferent at point of muscular disturbance, and the order renewed at the static battery where potential energy is converted into dynamic or kirsotic. Here then we have the complete cycles, one constant and persistent, the other, as it were, spasmodical or adventitious, governed by caprice of will. In involuntary muscular motion there exists a constant cycle, as in the case of the circulation there are two. There are two mural also humal cycles. Also in involuntary muscular motion of respiration there may be maintained two cycles, one mural the other aerial. In all the above cited instances there are cycles of motive power, and cycles of something moved, each and all complete and perfect in themselves. All these processes are dual, not mural in either case.

In all the above processes there has been a loss of energy in the entire organism, the energy so spent has been developed and sent up from all parts of the organism, every individual cell contributing its equivalent. As circulatory and respiratory motion are constant there is a constant exhaustion of nerve, or so called vital force, which has to be as constantly supplied by oxidation of all tissues and rebuilding of new by new protoplasm being periodically introduced. Every contraction and relaxation serves to exhaust or expend force or energy. It is well known that all energies in animal organisms are supplied from two sources, the organic kingdom and oxygen of the air meeting in the blood tide, first then in the tissues. Let us inquire into a paralized limb. We find here organic life alone and that of a low order, similating the vegetable more than the animal. All nerve energy is cut off and the life of the part even sustained only by the circulation, which is simply a mechanical process carried on through the paralized part by the force of the heart alone, and barely enough of life and nutrition to prevent mortification. Here again the part assimilates that of a cold blooded animal subject to changes of temperature from outside media to a certain degree, and the blood circulated in the part being constantly cooled by radiation of heat in passing through the limb, has a cooling depressing effect on the whole system. In this case nerve force or energy is cut off from the limb by some obstruction in nerve track, by which the will has no power to send or force beyond; this may be partial or complete.

Let us now examine into the nature of spasms. In such cases we find too much nerve or contractile energy let loose into the muscle which exerts a power on the contractile fibres much greater than normal, so much so that the muscle remains indefinitely contracted from some unknown cause, so much so that the will has no control, either in turning on or off this muscular force or even controlling it when on. This cramping or spasm of muscles is entirely independent of will, it being the same force that is sent by the will under ordinary circumstances. We may suppose that the irritating cause, whatever it may be or wherever located discharges all the will force that has been held in reserve by the will in a passive state. This spasm or cramp is a very exhaustive process, much more so than the will even after long and continued exertion in the most laborious manner. Over exertion of muscles sometimes causes cramps in them, whether from actual stunning of fibres, or over nutrition of the muscle serving to clog, thereby irritating. Irritation at one point may be transferred to distant muscles. In all these cases we have interrupted cycles abnormal, the demand being greater than the supply of systematic energy, hence prostration if long continued.

Sanctiviti or St Vitus's Dance is another form of nervous disorder when the muscles of volition are under the control of some disturbing cause in the nerves, or perhaps the muscles themselves may be involved, acting partly independent of the will and partly under its control, resulting in indefinite volition and mixed or indefinite cycle in such cases.

Other cases might be named. Such cases have pathological cycles. Pathological conditions also apply to involuntary muscular motion though not so frequent.

From what has been said and arguments advanced in my former articles, it becomes evident that all vital energies and forces are chiefly expended on muscular motion and and mainly on circulation and respiration which has to be kept up continually without interruption so long as life endures. The main object of this muscular involuntary motion seems to be to carry into the organism ternary and quarternary food together with oxygen, and carry out, in return the products of dissimilation or mineral elements. The chief object of all the above named processes seems to be to supply food for oxidation on the one hand and to furnish protoplasm or building material for the waste of cell structures. Organic compounds and oxygen enter the organism and mineral elements leave it, hence animals, especially of the higher order, do not subsist on mineral elements as these would not be so readily oxidizable, as organic combinations, otherwise minerals would serve the purpose of combustion and might take the place of the ternary group.

We find that the elements of man and the higher animals first called together from the mineral kingdom in the form of plants, where the binarys meet, are decomposed by the leaves and organic elements, from hence man is first plant and, possibly, hog, ox or sheep, then mineral in turn. Thus we find death taking place in every part of the organism at the same time, and when normal quantities and qualities of food are duly supplied, as constantly reproduced, otherwise starvation and general death follows. Thus we see spoke after spoke are removed from the wheels of life, and as constantly replaced by new ones, the joints, perhaps, less perfect each change of spoke, and life goes on taking down and rebuilding.

I shall not attempt to argue the question whether the body is a factor of the mind, or whether the mind is a factor of the organism. We may presume that mind is made up of cycles, as well as all motions in the cosmas organic and vital existence, forming two parts of the cosmas, hence animal motions form no exception to the general rule, even oxidation in the cells need not form an exception.

Life force then is a constant antagonism to the inorganic surroundings, the physical portion of the cosmas, and a constant struggle to a return to the primitive condition, or inorganic, where all life elements are in less complex combinations, or the binary condition. As no force is capable of self subordination the binary groups before metamorphosis, must be overcome by some new and more powerful energy.

This new energy may be supposed to be the organic breaking up of old and forming new and more complex with stronger affinities. Now, before the new affinities can in turn be overcome, energies still stronger than those that first overcame the binarys, must be brought to bear, and if brought about by the same forces these same forces must be removed and act in an opposite direction and with greater energy than in the first instance, resulting in resolution of elements. In other words no force or power can, without extrinsic interference, overcome or overturn itself. One great disturbing cause existing between the animal organism and the surrounding media, is difference in temperature, and the energetic struggle on the part of the organism to retain an elevated temperature some degrees higher with constant variations of the surrounding media, and the tendency to bring all bodies so influenced to its own temperature by natural interchanges of heat. Cold blooded animals cannot resist these outside influences to the same extent as the warm, owing to their slow oxidation and nutrition, thereby offering feebler resistance. When the temperature of the media reaches that of warm blooded animals, such animals are in greater danger of suffocation, from the fact that oxidation now becomes retarded to a certain extent and the antagonistics assuming more neutral relations, the consequence less disturbance. When the temperature rises above that of the animal body, the animal is then even in less danger, even cooling down the body with cooling drinks when the temperature becomes equal is advantageous, unless carried to excess; any difference is preferable to sameness of temperature, as the antagonisms are greater, and life cannot be sustained without them.

How muscular force is brought to bear on the fibre, or what portion of the muscle is acted upon, whether the whole at once or in parts in rapid succession, cannot be readily determined. It may be first spent in the centre of the fibrous mass, spreading in all directions, or it may be spent in some other mode, not known. It may invade the muscle from many or few points, at once entering and traversing in succession in wave circles How it enters or how it acts has not been fully determined, though one fact seems to apply significantly, that is, that there is a development of force, or accumulation in the muscle itself caused by the presence of the invasive muscular force. Or the mechanism of the muscular fibre may be such that a small force may exert immense power on mechanical principles. A slight touch of the trigger puts into motion a train of energies, the power and force of which are wholly disproportionate to the power so applied. The comparison is at least justifiable. On the present light and knowledge before us we are irresistibly drawn to the conclusion that the whole object of life is muscular motion and that all the energies of the animal organism are economically bottled up in reserve for the will which disturbs or flies the trigger that puts the muscular train in motion. All these forces may be summed up as cell activity, and that activity oxidation versus nutrition. Every cell may be supposed to be a separate life point or an integral part of the whole organism, having its own peculiar life force, though dependent on and subservient to all the balance, all mutually dependent, not one of which could be destroyed or greatly disturbed without reacting on all the balance, action and reaction being the order of life. All epithelial cells, if not oxidized, are cast off bodily from every surface in the body, and as constantly rebuilt bodily from the surface of the membrane basement or basement membrane by fissipuration or fissuration direct from the protoplasm. This species of cells being then bodily thrown off or shed, and as constantly replaced, not by slow disintegration, by the action of oxygen and rebuilt by integration of molecules as is the case with all other cells. Was it not for this constant desquamation of the skin, nerves and serous surfaces there would be an indefinite development going on outwardly from these surfaces that would be without limitation, and shapeless masses of flesh would be the result. But it has been otherwise ordered and certain definite limits fixed for the well being of the animal.

*To be Continued.*

---

